# Cardiovascular Autonomic Dysfunction in Hospitalized Patients with a Bacterial Infection: A Longitudinal Observational Pilot Study in the UK

**DOI:** 10.3390/biomedicines12061219

**Published:** 2024-05-30

**Authors:** Monica Arias-Colinas, Alfredo Gea, Joseph Kwan, Michael Vassallo, Stephen C. Allen, Ahmed Khattab

**Affiliations:** 1 Department of Preventive Medicine and Public Health, University of Navarra, 31008 Pamplona, Spain; 2 IdiSNA, Navarra Institute for Health Research, 31008 Pamplona, Spain; 3 Biomedical Research Network Center for Pathophysiology of Obesity and Nutrition (CIBEROBN), Carlos III Health Institute, 28029 Madrid, Spain; 4 Department of Brain Sciences, Imperial College, London W12 0NN, UK; 5 Department of Medicine for Older People, Royal Bournemouth Hospital, Bournemouth BH7 7DW, UK; 6 Faculty of Health and Social Sciences, Bournemouth University, Bournemouth BH8 8GP, UK

**Keywords:** autonomic nervous system, cardiovascular autonomic dysfunction, bacterial infection, cardiovascular abnormalities, heart rate variability

## Abstract

Purpose: A temporal reduction in the cardiovascular autonomic responses predisposes patients to cardiovascular instability after a viral infection and therefore increases the risk of associated complications. These findings have not been replicated in a bacterial infection. This pilot study will explore the prevalence of cardiovascular autonomic dysfunction (CAD) in hospitalized patients with a bacterial infection. Methods: A longitudinal observational pilot study was conducted. Fifty participants were included: 13 and 37 participants in the infection group and healthy group, respectively. Recruitment and data collection were carried out during a two-year period. Participants were followed up for 6 weeks: all participants’ cardiovascular function was assessed at baseline (week 1) and reassessed subsequently at week 6 so that the progression of the autonomic function could be evaluated over that period of time. The collected data were thereafter analyzed using STATA/SE version 16.1 (StataCorp). The Fisher Exact test, McNemar exact test, Mann–Whitney test and Wilcoxon test were used for data analysis. Results: 32.4% of the participants in the healthy group were males (*n* = 12) and 67.6% were females (*n* = 25). Participants’ age ranged from 33 years old to 76 years old with the majority being 40–60 years of age (62.1%) *(Mean age 52.4* SD *= 11.4*). Heart rate variability (HRV) in response to Valsalva Maneuver, metronome breathing, standing and sustained handgrip in the infection group was lower than in the healthy group throughout the weeks. Moreover, both the HRV in response to metronome breathing and standing up showed a statistically significant difference when the mean values were compared between both groups in week 1 (*p* = 0.03 and *p* = 0.013). The prevalence of CAD was significantly higher in the infection group compared to healthy volunteers, both at the beginning of the study (*p* = 0.018) and at the end of follow up (*p* = 0.057), when all patients had been discharged. Conclusions: CAD, as assessed by the HRV, is a common finding during the recovery period of a bacterial infection, even after 6 weeks post-hospital admission. This may increase the risk of complications and cardiovascular instability. It may therefore be of value to conduct a wider scale study to further evaluate this aspect so recommendations can be made for the cardiovascular autonomic assessment of patients while they are recovering from a bacterial infectious process.

## 1. Introduction

Autonomic dysfunction is the alteration of the normal autonomic nervous system function that has an adverse effect on an individual’s health [1]. It can present in a wide variety of ways depending on the etiology and whether it is associated with over- or under-activity [2]. Examples include orthostatic hypotension, excessive sweating, constipation, altered urinary frequency and erectile dysfunction [3,4]. Autonomic dysfunction can result from diseases that affect primarily either the CNS or the peripheral ANS [2]. The most common CNS-related autonomic dysfunction is degeneration of the intermediolateral cell columns (progressive autonomic failure) or disease/damage to the descending pathways that synapse on the intermediolateral column cells (spinal cord lesions, cerebrovascular disease, brainstem tumors, multiple sclerosis). The peripheral ANS can be damaged in acute and subacute autonomic neuropathies or in association with a generalized peripheral neuropathy, especially those involving damage to small fibers in the baroreflex afferent, the vagal efferents to the heart and the sympathetic efferent pathways to the mesenteric vascular bed [4,5].

The importance of assessing autonomic function and particularly cardiovascular reflexes is well documented in the literature. According to Kempler (2003), cardiovascular autonomic dysfunction (CAD) is a serious condition [6]. Since those reflexes play such a vital role in controlling cardiovascular functions such as blood pressure and heart rate, dysfunction of the cardiovascular autonomic reflexes has been linked to a higher risk of death [7,8,9]. In fact, there is evidence that links CAD to a higher risk of lethal arrhythmias and sudden death [6,10,11,12]. Particularly, it has been extensively reported that CAD is associated with higher mortality risk in individuals suffering from a range of conditions such as diabetes and cardiovascular disease [13,14,15,16] as well as in middle-aged and older individuals [11,17,18]. In addition to this, CAD is associated with infectious processes. Patients with HIV suffered from a degree of CAD [19,20]. Furthermore, CAD is common after Herpes simplex [21], leprosy [22], ‘Chagas’ disease [23] and rubella and Epstein–Barr virus [24]. Vassallo and Allen [25] showed a higher prevalence of impaired cardiovascular autonomic reflexes in the early recovery period after pneumonia compared to healthy individuals, and this improved significantly after 6 weeks, with a further improvement by 6 months. It is for these reasons that the assessment of the cardiovascular autonomic function is fundamental for clinical practice and the patient’s prognosis.

Although a number of tests have been proposed for the evaluation of the autonomic function, autonomic screening tests appear to focus mainly on the cardiovascular autonomic function as the results obtained from these tests are objective, quantifiable and well proven [26,27]. Moreover, tests of cardiovascular autonomic function can be used to identify individuals at risk of sudden death [7,8,9,28]. Heart rate variability (HRV) appears to be one of the best markers to assess cardiac autonomic function [12,29,30,31]. HRV is the measurement of the temporal variability between heartbeats in relation to the mean heart rate [32,33] and provides information on the sympathetic and parasympathetic balance of the autonomic nervous system (ANS) [33,34] particularly, cardiac vagal tone [29,35]. 

As has been discussed previously in this section, CAD appears to be a common finding during and after certain infectious processes. However, most of the research on this topic focuses on viral conditions, and therefore, the results may not be applicable to bacterial infections. Bacterial infections are extensively embedded in society. They remain a major cause of suffering and death, and the mortality rate due to infective processes appears to remain high [36]. Over the years a wide range of literature has focused on trying to increase the effectiveness of the strategies used to diagnose and treat bacterial infections [37]. It is in this context that the study of CAD by means of the HRV and its link to bacterial infective processes appears to be an important line of research to find out whether an altered HRV is associated with infection and its prognosis [38]. Hence, this research aimed to explore the prevalence of CAD in the recovery period from a bacterial infection.

## 2. Material and Methods

### 2.1. Study Design

An exploratory longitudinal observational pilot study was conducted to explore the prevalence of CAD during the recovery period of an infection. The study was conducted in a blinded setting, and it was approved by the Local Research Ethics Committee. Each patient received verbal information about the study, a patient information sheet, and a consent form. Patients were assured that their participation was anonymous and would not interfere with their medical treatment and that they could withdraw from the study at any time. The study was conducted according to the principles stated in the Declaration of Helsinki.

### 2.2. Participants and Sample: Inclusion and Exclusion Criteria

Two groups of patients were selected: the infection group and the healthy group.

The general inclusion criteria were (1) older than 18 years old and able to freely agree to participate; (2) participants needed to be clinically stable by the time the first cardiovascular autonomic assessment was carried out (week 1 from hospital admission); (3) participants needed to be able to satisfactorily perform the autonomic function tests.

For the infection group, hospitalized patients with a diagnosis of an active and symptomatic bacterial infection were recruited. Patients in this group had pneumonia, other respiratory tract infections, urinary tract infections, pyelonephritis or cellulitis. Patients with sepsis were excluded from participating in the study as it was considered they might not meet the criteria of hemodynamic stability required at the time of the first cardiovascular autonomic function assessment (within a week from hospital admission). Moreover, patients whose infections were hospital-acquired were excluded because this group of patients was more likely to be particularly ill or immunosuppressed, which could therefore introduce bias into the autonomic function assessment results. All eligible patients were included regardless of when the infection started, as long as the patient was currently symptomatic and required inpatient treatment. The criteria of stability were the patient being adequately hydrated with improving biochemical profile, apyrexial (T^a^ < 37.8 °C), systolic blood pressure > 90 mmHg, heart rate ≤ 100 beats/minute, respiratory rate ≤ 20 respirations/minute and pulse oximetry ≥ 90%, treatment started and well enough to participate in the assessment protocol [39,40].

The exclusion criteria were (1) patients with significant communication difficulties (e.g., severe aphasia); (2) patients with severe cognitive impairment (mini-mental state examination 24 or less); (3) patients who were unable to stand or hold their breath to perform the Valsalva maneuver; (4) patients who had a past history of, or active vascular or cardiovascular problems such as myocardial infarction, coronary artery disease, heart failure and stroke as the HRV of these patients could already be compromised; (5) patients with Parkinson’s disease; (6) diabetic individuals; (7) patients with known autonomic dysfunction or orthostatic hypotension; (8) patients with depression; (9) patients who were on drugs known to affect the autonomic function such as beta blockers and antidepressants. Those patients whose infections were hospital-acquired were also excluded because this group of patients was more likely to be particularly ill or immunosuppressed, which could therefore introduce bias into the autonomic assessment results. Patients who were seriously ill or were deemed unlikely to recover sufficiently to take part in the study within the subsequent 6 weeks were not approached.

Then, the healthy group was selected. Individuals in this group had not had a bacterial infection requiring treatment for the previous 6 months. Participants in this group were selected with age and sex as similar as possible to the infection group.

### 2.3. Study Protocol and Data Collection

**Figure 1:**
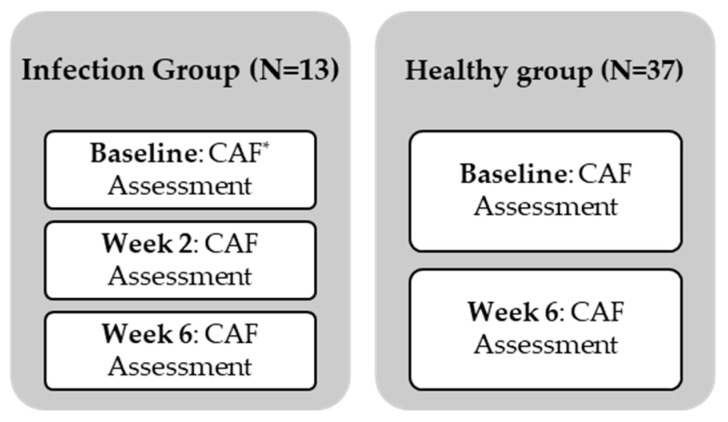
Study protocol and data collection points for both the infection and the healthy group. * CAF: Cardiovascular Autonomic Function assessment: Evaluation of HRV in response to Valsalva, deep breathing, standing up and sustained handgrip.

The baseline autonomic function assessment was carried out at the beginning of the study in the healthy group and thereafter at the follow up visits after 6 weeks. In the infection group, three assessments were performed throughout a 6-week period (weeks 1, 2 and 6) (Figure 1). Cardiovascular autonomic assessment was based on the widely used Ewing method with minor modifications [41]. The acquisition system used a multi-channel biosensor manufactured by Procomp Infinity, with an ECG, photoplethysmography and chest wall movement sensor. The recording was at a very fast sampling rate of 2048 Hz. The standard Ewing bedside tests were augmented by an extended period of metronome-guided breathing at 6 breaths per minute and the quantification of the variation in the HR in response to sustained handgrip. The latter acquisition process requires no more than 5 min more than the conventional Ewing and Clarke method.

Participants were asked to have a light breakfast and refrain from smoking or drinking coffee and alcohol for at least 2 h before performing the test. Furthermore, all participants were asked to adopt a 30 to 45 degrees tilt position. All participants’ appointments were made during the morning or early hours of the afternoon. In addition to this, participants were all assessed in a quiet room with a comfortable temperature (23 °C approximately). Participants were asked to rest for 10 min before commencing the tests in an attempt to obtain baseline conditions for the heart rate.

The following sequence of tests was used to assess cardiac autonomic reflexes:

1. HRV in response to Valsalva Maneuver. For this test, the patient needed to blow into a mouthpiece attached to a mercury barometer at a minimum expiratory pressure of 40 mmHg for 10 to 15 s while the ECG was being recorded [3,42,43,44].
2. HRV in response to metronome breathing: the patient was asked to breathe deeply and steadily at a rate of 6 breaths per minute for 2 min while the HR was being recorded by means of an ECG [45,46].
3. HRV in response to standing up: for this test, the patient changed his position from lying down to standing up while the HRV was being assessed by means of an ECG.
4. HRV in response to sustained handgrip: For this assessment, handgrip was maintained around at least 30% of the person’s maximum handgrip for 3 to 5 min [46] while the HRV was monitored.

### 2.4. Statistical Analysis

Data were analyzed using STATA/SE version 16.1 (StataCorp), two-sided *p*-values were used and the statistical significance threshold was set a priori at 0.05. The percentage of participants with an abnormal autonomic function was compared between the healthy and infected groups by means of the Fisher exact test. This test was used as each of the autonomic function evaluations was independent, and the number of expected values was expected to be <5. Furthermore, the McNemar exact test was used to evaluate whether the percentage of participants with an abnormal autonomic function improved throughout the weeks (week 1 to week 6) within each of the groups (healthy and infected). This statistical test was used as the aim was to compare the autonomic function of the same groups at two different points of time to evaluate if there was a within-group change (paired samples). The mean values of each of the HRV parameters were compared between the healthy and infection groups by using the Mann–Whitney test. Moreover, the Wilcoxon test was applied to evaluate if the mean value of HRV parameters improved throughout the weeks within each of the groups. These tests (Mann–Whitney and Wilcoxon) were used as they are nonparametric tests, which were deemed to be the most appropriate considering the small sample size of this study. 

## 3. Results

### 3.1. Characteristics of Participants

Fifty participants met the inclusion criteria and were enrolled in this study. There were 37 and 13 participants in the healthy group and infection group, respectively. Of the participants, 32.4% in the healthy group were males (*n* = 12), and 67.6% were females (*n* = 25). Participants’ age ranged from 33 years old to 76 years old, with the majority being 40–60 years of age (62.1%) *(Mean age 52.4* SD *= 11.4*). Of the 13 individuals in the infection group, 7 were males (53.8%), and 6 were females (46.2%). Furthermore, 38.5% of the individuals in this infection group were below the age of 40, and 30.8% were aged 60 to 70 years old, with a mean group age of 47.9 (Table 1). Furthermore, all participants in the two groups were independent and fully mobile. Six participants (16.2%) in the healthy group were smokers, three (8.1%) of whom smoked less than 10 cigarettes per day (8.1%), one individual (2.7%) smoked 15 cigarettes and two (5.4%) smoked more than 20 cigarettes per day. Furthermore, 32.4% (*n* = 12) of participants in this group did not drink any alcohol, and the same number of individuals confirmed that they drank less than 5 units per week. On the other hand, 5.4% (*n* = 2) of individuals drank more than 15 units per week. Moreover, five (38.5%) individuals in the infection group were smokers, 3 (23%) of whom smoked 10 to 20 cigarettes per day, one (7.7%) smoked less than 10 cigarettes per day and one (7.7%) smoked over 20 cigarettes per day. Most participants in the infection group drank none (30.7%) or 1 to 5 units of alcohol per week (38.5%) (Table 1).

The initial assessment conducted on admission to the hospital showed that the oxygen saturations of the infection group ranged from 89% to 100%, with a group mean saturation of 95.9%. One individual in this group (7.7%) was hypoxic upon hospital admission, with oxygen saturation below 90%. In addition to this, one individual (7.7%) was hypotensive; the SBP ranged from 86 mmHg to 155 mmHg and the DBP ranged from 43 mmHg to 100 mmHg (*Mean* SBP 120.6 mmHg; SD= 19.7 and *Mean* DBP 64.4; SD = 15.0). The temperature and the HR were also measured on admission, and these measurements showed that the tympanic temperature ranged from 36.2 °C to 39.7 °C, with a mean temperature of 38.1 °C. Eight (61.5%) individuals were pyrexic on admission. Additionally, whereas no individuals were bradycardic, 38.5% of the participants in the infection group were tachycardic.

Most participants in the infection group were admitted to the hospital with pneumonia (38.5%, *n* = 5). One individual suffered from a urinary tract infection and another one with cellulitis. In addition to this, 46.15% (*n* = 6) presented with different types of infections, including lower respiratory tract infection, infective exacerbation of chronic obstructive pulmonary disease, infective exacerbation of asthma and pyelonephritis. All of them were discharged from the hospital before week 6 of follow up.

### 3.2. Cardiovascular Autonomic Function 

The cardiovascular autonomic function was assessed in the healthy group, and the results showed that the great majority of individuals in this group presented with normal HRV in response to all protocol stimuli both in week 1 and week 6 (Table 2). On the other hand, the HRV of the participants in the infection control group showed a different trend, as the results revealed that 40% (*n* = 4) of individuals in the infection group had an abnormal HRV in the first visit. This, however, improved slightly by week 6 as 33.3% (*n* = 3) had an abnormal HRV (Table 2). Furthermore, there was a statistically significant difference between the proportion of individuals with abnormal cardiovascular autonomic function in between the groups in week 1 (*p* = 0.018) and a trend towards significance in week 6 (*p* = 0.057) (Table 2). Moreover, there was no evidence of an improvement in the proportion of patients with abnormal autonomic function (*p* > 0.999) in the infection group (Table 2).

**Figure 2:**
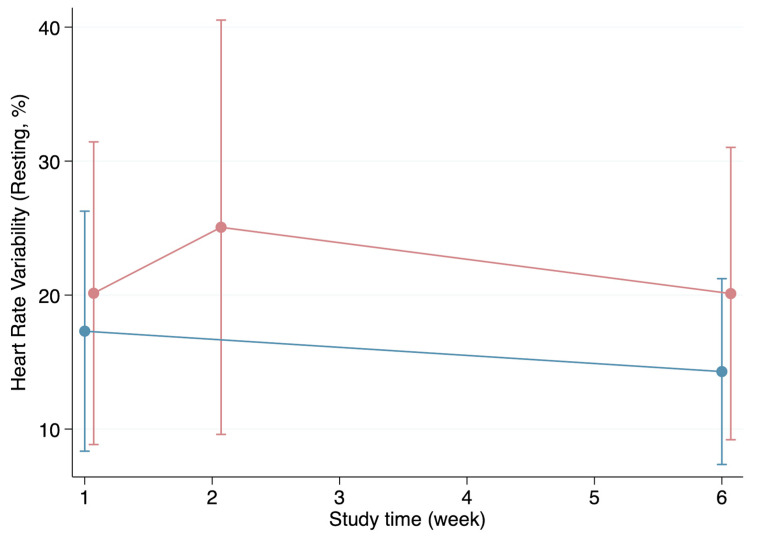
Progression of the heart rate variability (resting) throughout the weeks in the healthy (blue line) and infection (red line) groups (mean and standard deviation).

**Figure 3:**
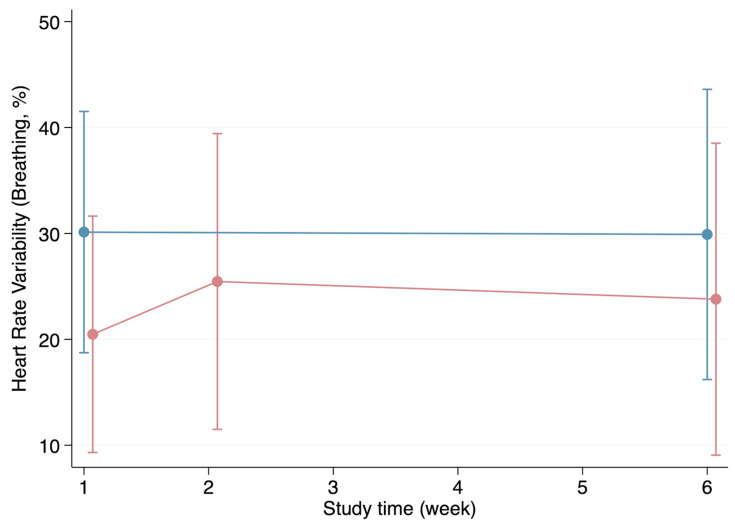
Progression of the heart rate variability (breathing) throughout the weeks in the healthy (blue line) and infection (red line) groups (mean and standard deviation).

**Figure 4:**
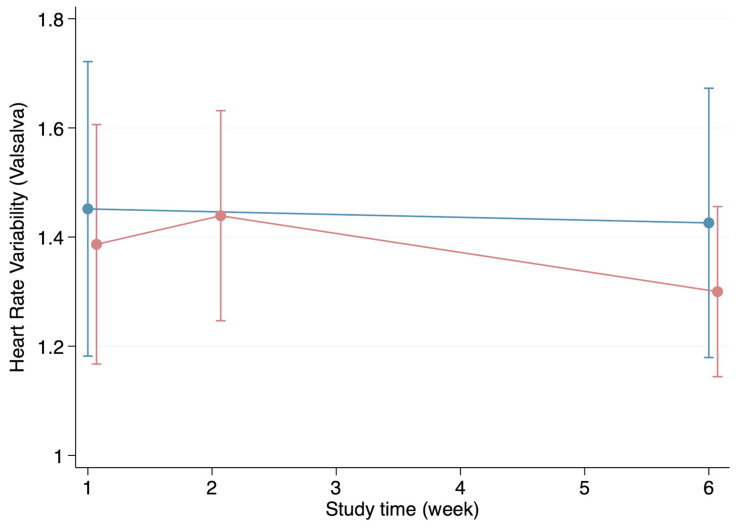
Progression of the heart rate variability (Valsalva) throughout the weeks in the healthy (blue line) and infection (red line) groups (mean and standard deviation).

**Figure 5:**
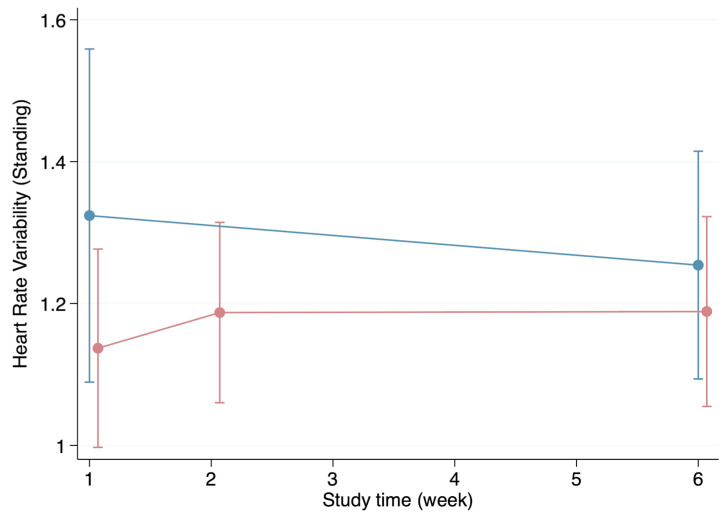
Progression of the heart rate variability (standing) throughout the weeks in the healthy (blue line) and infection (red line) groups (mean and standard deviation).

The HRV appeared to be generally higher in the healthy group during the first visit for all parameters except for the resting HRV. Likewise, the individuals in the healthy group presented with higher HRV values during week 6 except, once again, for the resting HRV (Figure 2, Figure 3, Figure 4 and Figure 5). Both the HRV in response to metronome breathing and standing up showed a statistically significant difference when the mean values were compared between both groups in week 1 (*p* = 0.03 and *p* = 0.013) (Table 3).

Moreover, the HRV results at the first visit were compared with the HRV results at the last visit within each of the groups to evaluate whether the HRV changed over time (Table 3). These within-group comparisons showed that the HRV values in the healthy group did not change significantly from the first visit to the last one. Furthermore, although the mean HRV generally improved from week 1 to week 6 in the infection group, these improvements were not statistically significant (Table 3).

Percentages of patients with abnormal cardiovascular autonomic function are presented, along with the number of patients with abnormal cardiovascular autonomic function over the total number of patients where enough information was available to be able to reach that conclusion. For the change in the proportion of patients with abnormal cardiovascular autonomic function, only those patients with information in both assessments were used (*n* = 31 and *n* = 8, respectively).

## 4. Discussion

For this study, the cardiovascular autonomic function of individuals with an acute bacterial infection was assessed over a 6-week period. Moreover, the autonomic function was also evaluated in a group of healthy individuals so that comparisons could be made. The evaluation of the HRV was used for the assessment of the cardiovascular autonomic function in both groups, as suggested by Ewing and Clarke [41]. The aim was to find out if individuals admitted to the hospital with a bacterial infection presented with a lower HRV and therefore more abnormal than healthy individuals. 

The results of this study showed that the cardiovascular autonomic function of healthy individuals was generally normal throughout the weeks, which was the expected finding considering that this group was composed of individuals with no apparent health problems at the time of the data collection. This was, however, not the case when evaluating the HRV in the infection group. The cardiovascular autonomic function of a proportion of individuals with an acute bacterial infection was impaired during the first data collection point (week 1), and, interestingly, there was no evidence of an improvement in the autonomic function throughout the six-week period for this infection group (Table 2).

This was a surprising finding considering that it had been suggested in the literature that if a CAD was present, an improvement should have been appreciated after 6 weeks from infection [25]. Ahmad et al. [47] agreed with Vassallo and Allen [25] and suggested that the HRV of individuals with sepsis would decrease during the acute phases of the infection but would start recovering as soon as antibiotic therapy and other rectifying measures were implemented. Ahmad et al. [47] did not, however, provide a clear timeline whereby recovery of the HRV post-infection should be expected. Furthermore, no other research has been found to clarify this aspect, and therefore, it is not possible to actually establish when the cardiovascular autonomic function post-infection should return to normal. However, it seems plausible to consider that, as Ahmad et al. [47] suggest, a lower HRV in the presence of infection should return to normal when the infection is treated and the individual recovers from it. However, in this study, CAD was still prevalent after 6 weeks from infection onset. By this time, all individuals in this infection group had received antibiotic therapy, they were hemodynamically stable and at home and subsequently, the HRV should have returned to its normal values. It could be argued that the individuals in this infection group with an autonomic dysfunction could be suffering from other conditions that might affect the autonomic cardiovascular reflexes and were not diagnosed yet or were not considered as an exclusion criterion for this project. Although the exclusion criteria were exhaustive and included all those conditions that are known to affect the HRV, such as cardiovascular disease [48], Parkinson’s disease [49], and diabetes [50], it is recognized that the list of conditions cannot be conclusive as there may be conditions which affect the ANS and there is yet no evidence to demonstrate so. In addition to this, it may be possible that individuals in this group may have been suffering from a clinical condition at the time of the data collection point, which they failed to disclose as they did not deem it necessary or had not been diagnosed (chronic inflammatory condition for example). This would be in agreement with what the evidence shows as some chronic inflammatory conditions such as rheumatoid arthritis, lupus disease, chronic inflammatory joint disease, and inflammatory bowel disease are associated with an early appearance of autonomic dysfunction, which may be present even before the classical symptoms of those conditions manifest themselves [33,51,52,53,54]. Furthermore, the abnormal CAD could be due to the presence of an underlying viral infection, which they did not disclose or they were not even aware of. As has been previously discussed in this work, there is evidence that suggests that CAD is common in the context of viral infections [21,24]. 

It is for these reasons that it is difficult to fully justify or comprehend why the autonomic function was still abnormal in one-third of individuals in week 6. Furthermore, it can be suggested that a longer follow up appointment would have led to further improved cardiovascular autonomic function, as suggested by Vassallo and Allen [25]. Further research would therefore be recommended to fully understand the recovery pattern of cardiovascular autonomic function post-bacterial infection.

### Limitations of the Study

This study was set to explore the cardiovascular autonomic function in the context of acute bacterial infections. There were a number of exclusion criteria that needed to be considered to ensure the obtained results were as much as possible due to the actually conducted assessments and not to other confounding factors. The exclusion criteria were exhaustive and evidence-based. However, they included a number of conditions that are very prevalent in today’s society, which narrowed the study population enormously. Conditions such as diabetes, Parkinson and heart disease are very prevalent in society, and a large number of the individuals admitted to the hospital with a bacterial infection would suffer from any of those conditions and would not be eligible to participate as their autonomic function could already be compromised. Moreover, the exclusion criteria included a list of drugs that can potentially affect the individuals’ autonomic function. The list included very common medications such as beta blockers, alpha blockers and opioids and a number of potential participants had to be excluded on those grounds. Only a very small number of individuals were eligible to participate and were approached by the researcher to be invited to the study. Consequently, the sample size was small. Although this was a pilot study, the small sample size may have affected the interpretation and the validity of the obtained results. It is therefore for this reason that in the future, a larger scale study can be carried out to further explore these issues in greater detail. 

Another aspect that may have limited this pilot study was that the follow up appointments may not have been separated long enough for the significant changes in the autonomic function to be detected. Although there is no clear timeline to determine when the cardiovascular autonomic function during the recovery period from a bacterial infection should return to normal, some authors state that it should improve 6 weeks post-onset of acute bacterial infection, and a further improvement would be detected 6 months post-infection [25]. It is therefore reasonable to consider that a follow up after 6 months could have produced different and perhaps more significant results when assessing the autonomic function of the individuals in this study. However, as this was a pilot study, a follow up after 6 months may not have been a realistic goal that could have been achieved. It is therefore suggested that further research needs to be conducted on the cardiovascular autonomic function post-acute bacterial infection, where further time is allowed between follow ups in an attempt to identify further changes or possible improvements. However, if longer follow ups are planned, it is anticipated that other factors could appear in that period of time (such as newly diagnosed conditions or clinical events), and that would need to be considered as they could potentially interfere with the cardiovascular autonomic function of individuals. 

## 5. Conclusions 

The results of this study suggest that CAD, as assessed by the HRV, is a common finding during the recovery period of a bacterial infection even after 6 weeks post-hospital admission. This autonomic dysfunction may increase the risk of complications and cardiovascular instability. Subsequently, the assessment of the cardiovascular autonomic function of individuals may be required while recovering from a bacterial infection so that possible complications can be promptly identified. Although this is a pilot study, the results from this work may serve as the basis for further research on the evaluation of the cardiovascular autonomic function and its recovery pattern post-bacterial infection. Future research projects should aim at further exploring this phenomenon and its evolution so that recommendations for practice can be made.

## Figures and Tables

**Table 1:** Details of patient groups.

	Healthy Group	Infection Group
Number	37	13
Mean age (years)	52.4	47.9
Range	(33–76)	(24–69)
Females	67.6% (25/37)	46.2% (6/13)
Males	32.4% (12/37)	53.8% (7/13)
Smokers	16.2% (6/37)	38.5% (5/13)
Alcohol > 14 Units/week	5.4% (2/37)	7.7% (1/13)
Independent with ADLs *	100% (37/37)	100% (3/3)
Mean White blood cells × 10^9^/L (SD) ^†^	-	15.4 (5.9)
Mean CRP mg/L (SD) ^†^	-	145 (107)
Mean Oxygen sat % (SD) ^†^	-	96 (3)
Pyrexia °C (%) ^†^	-	61.5% (8/13)

* ADLs—activities of daily living. ^†^ Parameters on hospital admission.

**Table 2:** Percentage of participants with abnormal cardiovascular autonomic function healthy group and infection group, first and last visit.

	Week 1	Week 6	Within Group Difference of % (95% CI; *p*-Value of Change) **
Healthy group	5.88% (2/34)	6.06% (2/33)	0% (−12.2 to 12.2%; *p* > 0.999)
Infection group	40% (4/10)	33.33% (3/9)	0% (−61.5 to +61.5%; *p* > 0.999)
Between groups difference of % (*p*-value) *	+34.12% (2.7 to 65.5%; *p* = 0.018)	+27.3% (4.6 to 59.1%; *p* = 0.057)	

* Fisher Exact Test; ** McNemar Test.

**Table 3:** Summary of results of autonomic function tests.

		Week 1	Week 6	Within Group Difference (*p*-Value) **
HRV Valsalva	Healthy group	1.45 (0.27)/36	1.43 (0.25)/32	−0.018 (−0.113 to 0.076; *p* = 0.76)
	Infection group	1.39 (0.22)/9	1.30 (0.15)/9	−0.096 (−0.313 to 0.121; *p* = 0.30)
	Between groups difference (95% CI; *p*-value) *	−0.065 (−0.261 to −0.131; *p* = 0.62)	−0.123 (−0.298 to 0.053; *p* = 0.203)	−0.077 (−0.292 to 0.138; *p* = 0.36)
HRV Breathing	Healthy group	30.13 (11.39)/35	29.91 (13.70)/35	−0.08 (−3.885 to 3.725; *p* = 0.81)
	Infection group	20.47 (11.16)/11	27.31 (17.36)/9	+5.7 (−3.65 to 15.05; *p* = 0.15)
	Between groups difference (95% CI; *p*-value) *	−9.652 (−17.550 to −1.754; *p* = 0.03)	−2.594 (−13.507 to −8.319; *p* = 0.53)	+5.62 (−3.080 to 14.320; *p* = 0.39)
HRV Handgrip	Healthy group	1.36 (0.24)/34	1.28 (0.16)/31	−0.073 (−0.182 to 0.037; *p* = 0.38)
	Infection group	1.28 (0.15)/10	1.46 (0.52)/9	+0.07 (−0.012 to 0.152; *p* = 0.11)
	Between groups difference (95% CI; *p*-value) *	−0.745 (−0.237 to 0.088; *p* = 0.52)	+0.182 (−0.031 to 0.395; *p* = 0.36)	+0.143 (−0.065 to 0.350; *p* = 0.09)
HRV Standing	Healthy group	1.32 (0.23)/35	1.25 (0.16)/34	−0.067 (−0.163 to 0.029; *p* = 0.31)
	Infection group	1.14 (0.14)/10	1.18 (0.13)/9	+0.053 (−0.079 to 0.184; *p* = 0.57)
	Between groups difference (95% CI; *p*-value) *	−0.187 (−0.345 to −0.029; *p* = 0.013)	−0.074 (−0.191 to 0.043; *p* = 0.16)	+0.119 (−0.081 to 0.320; *p* = 0.25)

* Mann–Whitney test; ** Wilcoxon Test. Numbers are mean (standard deviation)/number of patients with information or difference (*p*-value). For within-group differences, only patients with information at both time assessments were included.

## Data Availability

The raw data supporting the conclusions of this article will be made available by the authors on request.
